# *Komagataella phaffii* Erp41 is a protein disulfide isomerase with unprecedented disulfide bond catalyzing activity when coupled to glutathione

**DOI:** 10.1016/j.jbc.2024.105746

**Published:** 2024-02-13

**Authors:** Arianna Palma, Lukas A. Rettenbacher, Antti Moilanen, Mirva Saaranen, Brigitte Gasser, Lloyd W. Ruddock

**Affiliations:** 1 Department of Biotechnology, Institute of Microbiology and Microbial Biotechnology, University of Natural Resources and Life Sciences (BOKU), Vienna, Austria; 2 Austrian Centre of Industrial Biotechnology, Vienna, Austria; 3 School of Biosciences, University of Kent, Canterbury, UK; 4 Faculty of Biochemistry and Molecular Medicine, University of Oulu, Oulu, Finland

**Keywords:** disulfide, endoplasmic reticulum (ER), enzyme catalysis, glutathione, protein disulfide isomerase, protein folding, yeast, unfolded protein response (UPR)

## Abstract

In the methylotrophic yeast *Komagataella phaffii*, we identified an endoplasmic reticulum–resident protein disulfide isomerase (PDI) family member, Erp41, with a peculiar combination of active site motifs. Like fungal ERp38, it has two thioredoxin-like domains which contain active site motifs (*a* and *a’*), followed by an alpha-helical ERp29c C-terminal domain (*c* domain). However, while the *a* domain has a typical PDI-like active site motif (CGHC), the *a’* domain instead has CGYC, a glutaredoxin-like motif which confers to the protein an exceptional affinity for GSH/GSSG. This combination of active site motifs has so far been unreported in PDI-family members. Homology searches revealed ERp41 is present in the genome of some plants, fungal parasites, and a few nonconventional yeasts, among which are *Komagataella* spp. and *Yarrowia lipolytica*. These yeasts are both used for the production of secreted recombinant proteins. Here, we analyzed the activity of *K. phaffii* Erp41. We report that it is nonessential in *K. phaffii*, and that it can catalyze disulfide bond formation in partnership with the sulfhydryl oxidase Ero1 *in vitro* with higher turnover rates than the canonical PDI from *K. phaffii*, Pdi1, but slower activation times. We show how Erp41 has unusually fast glutathione-coupled oxidation activity and relate it to its unusual combination of active sites in its thioredoxin-like domains. We further describe how this determines its unusually efficient catalysis of dithiol oxidation in peptide and protein substrates.

Disulfide bonds primarily stabilize the mature structure of proteins and guide the folding of nascent polypeptide chains through conformational space. A large number of proteins contain disulfide bonds, including structural proteins and biopharmaceutical targets such as antibodies. As disulfide intermediates can be formed by any two cysteines, reaching the native arrangement is often rate limiting both *in vitro* and *in vivo* (1, 2). Disulfides can be generated spontaneously by exposure of thiol groups to molecular oxygen, but such slow and random reactions cannot account for the protein folding requirements of a cell, therefore enzymatic machineries have evolved to facilitate this process. Bacteria restrict disulfide bond formation to the periplasm where it occurs with the assistance of the Dsb enzyme pathway. In eukaryotes, disulfide bond formation mainly takes place within the endoplasmic reticulum (ER), where protein disulfide isomerases (PDIs) oxidize and isomerize exposed thiols of nascent polypeptides in partnership with sulfhydryl oxidases, such as Ero1, and low molecular weight compounds such as reduced (GSH) and oxidized (GSSG) glutathione and peroxides (3, 4, 5).

PDIs belong to the thioredoxin superfamily. Canonical PDI exhibits a flexible horseshoe structure comprised of catalytically active (*a*) and inactive (*b*) domains each organized as a core of four to five stranded β-sheets flanked by α-helices (6, 7, 8). Thioredoxin active sites present a -CXXC- consensus motif, where the nature of the residues between the cysteines is known to affect the redox potential and, ultimately, catalysis (9). The thioredoxin superfamily hosts a range of enzymes that can act as reductases, oxidases, isomerases, GSH S-transferases, peroxidases, or glutaredoxins (Grxs) (6, 10). The redox potential, stability of the disulfide-dithiol active site states, and substrate specificity define their main activity (11, 12, 13). For instance, PDIs typically exhibit a -CGHC- motif, in which the histidine stabilizes spatially adjacent thiolate anions, causing instability to the disulfide bond, and thus favor catalysis of dithiol oxidation in substrate proteins (14). In contrast, Grxs present -CX(Y/F)C- in their active sites, where the aromatic sidechains form part of the GSH binding groove (15, 16).

In the past decades, approximately twenty different PDI family proteins have been identified in the mammalian proteome (17, 18). Human PDIA1 (hPDI) and its homolog Pdi1 in the yeast *Saccharomyces cerevisiae* are by far the most studied PDI family members both *in vitro* and *in vivo,* and it is thought to be the main catalyst for disulfide formation and isomerization (18). It is essential in mammals (19) and yeast, where *pdi1*Δ mutants were shown to be lethal (20). Other PDI family members are thought to have more specialized functions. For example, among human paralogs, ERp57 was found to be responsible for the folding of glycoproteins (21), while other members, such as ERp27, ERdj5, or ERp90, are involved in the unfolded protein response (22, 23, 24). However, the physiological contribution of most of these enzymes during different phases of the cell cycle or in different cell or tissue types is still an open research topic. Furthermore, comparative predictions at the sequence level are usually performed to infer the function by homology for PDI family members from nonmodel organisms.

The methylotrophic yeast *Komagataella phaffii* (syn *Pichia pastoris*) is a popular host for the production and secretion of industrial and biopharmaceutical protein targets (25). Recently we have detailed the kinetics of the *K. phaffii* Ero1–Pdi1 axis for disulfide formation and showed that it presents novel mixed biochemical features between the *S. cerevisiae* and human systems (26). In addition to Pdi1, *K. phaffii* encodes four more PDI family members according to sequence-based predictions: Mpd1 (PP7435_Chr1-0128), Eps1 (PP7435_Chr2-0878), an ERp38 family member with unusual active sites (PP7435_Chr1-0470), and one uncharacterized protein localized on genomic locus PP7435_Chr4-0138 (17) All PDI family members except for the uncharacterized protein PP7435_Chr4-0138 are found in the ER proteome under standard growth conditions (27, 28). While Pdi1, Mpd1, and Eps1 are also present in *S. cerevisiae*, ERp38 family members are not. Furthermore, while previously reported ERp38 family members (29, 30) have the usual PDI active site motif (CGHC), the one in *K. phaffii* has an unusual combination of active site motifs. From sequence analysis this PDI would be expected to have hybrid characteristics between PDIs and Grxs, something that has never been characterized to date in PDI family members.

Here, we purify and characterize the *K. phaffii* ERp38 family member, which we name Erp41, and demonstrate that it has distinct enzymological properties, which include an unprecedented rate of disulfide bond formation when coupled to a GSH buffer.

## Results

### *K. phaffii* Erp41 is a unique, nonessential PDI-family member

The *K. phaffii* ERp38 PDI family member encoded by PP7435_Chr1-0470 is to date uncharacterized at the protein level. Based on its three-dimensional fold and domain structure (Fig. S1), with two catalytic *a* domains (*a*-*a’*), no noncatalytic thioredoxin-fold *b* domains and a C-terminal ERP29_C α-helical bundle, it resembles ERp38 (also known as TigA) from *Aspergillus niger* and *Neurospora crassa* (29, 30) and was originally named ERp38 (17). However, and in contrast to *A. niger* and *N. crassa* ERp38, which have two CGHC active site motifs, primary structure analysis revealed that *K. phaffii* PP7435_Chr1-0470 has two unequal active site motifs: -CSHC- (in the *a* domain) and -CGYC- (in the *a’* domain). The former motif is PDI-like, while the latter is Grx-like. As this would be expected to give the protein a distinct activity, we chose to name this ER protein with 41 kDa as Erp41, an ERp38 family member. When considering such unequal active sites as criteria, Erp41 sequence homologs can be found in relatively few organisms. This includes few members of the *Saccharomycetes* (*e.g., Komagataella pastoris, Komagataella kurzmanii, Yarrowia lipolytica*) and *Schizosaccharomycetes* (Table S1). Notably, ERp38-like proteins are absent in the well-characterized yeast *S. cerevisiae* and closely related species. Erp41 is also not found in other methylotrophic yeasts such as *Ogataea* spp. that are phylogenetically close to *K. phaffii*. While all thioredoxin superfamily members share a similar fold, no known characterized member of the PDI family that we are aware of shows such a mixture of active sites. Therefore, we wondered whether Erp41 was enzymatically closer to PDIs or Grxs and what its role in disulfide formation in the ER might be.

We first investigated whether Erp41 was essential in *K. phaffii*. Therefore, the gene was fully knocked out by CRISPR-Cas9 homology directed recombination. Several transformants with the correct PCR-verified knock out were obtained (Fig. S2), suggesting that Erp41 is nonessential in standard growth conditions. This was further supported by growth experiments on solid and liquid media, where no phenotypic differences between *Erp41*Δ and the WT strain were noticed in defined or rich media, even when supplemented with folding interferents, such as tunicamycin, DTT, and diamide (Fig. S3, *A* and *B*).

### Erp41 coupled to Ero1 and GSH has faster oxygen consumption kinetics than Pdi1

To define the function of Erp41, we produced it in a heterologous expression system, purified it and analyzed it biophysically and enzymatically. The purified protein had the correct mass (Table S2), exhibited a CD spectrum (Fig. S4*A*), consistent with the expected fold predicted by AlphaFold (Fig. S1) and had a midpoint for thermal denaturation of 42 °C (Fig. S4*B*).

**Figure 1:**
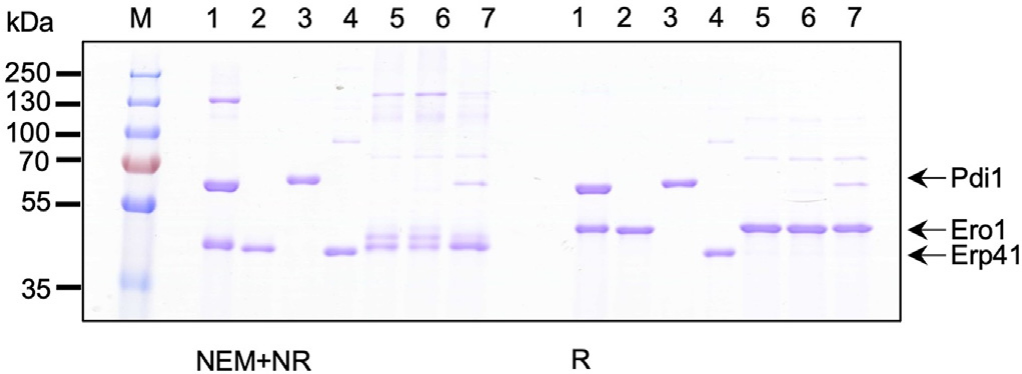
**Small-scale coexpression tests of PDI-family members with *Komagataella phaffii* Ero1.** SDS-PAGE showing small-scale expression and immobilized metal affinity chromatography purification of (1) coexpression of Ero1 and Pdi1 in the presence of *Kp*PDI CyDisCo (26), (2) expression of Ero1^C136A^ in the presence of *Kp*PDI CyDisCo, (3) expression of Pdi1, (4) Erp41, (5) co-expression of Erp41 and Ero1, (6), coexpression of Erp41 and Ero1 in the presence of classical human PDI CyDisCo (50), (7) co-expression of Erp41 and Ero1 in the presence of *Kp*PDI CyDisCo (26). On the *left side* of the gel, NEM-blocked nonreduced samples are shown, while, on the *right side*, reduced samples are shown in the same order. An uncropped image of the gel is provided in Fig. S5. NEM, N-ethylmaleimide; PDI, protein disulfide isomerase.

Next, we analyzed the potential interaction of Erp41 with *K. phaffii* Ero1. As recently discovered (26), in *K. phaffii,* inactive Ero1 is bound to Pdi1 *via* an intermolecular disulfide, involving Cys136 on Ero1 and Cys404 in the *a’* domain of Pdi1. Thus, we initially examined if Erp41 could functionally replace Pdi1 in this complex. As previously reported (26), cytoplasmic expression of *K. phaffii* Ero1 and Pdi1 in *Escherichia coli* CyDisCo assisted by the coexpression of Erv1p for disulfide formation allowed isolation of a complex of *K. phaffii* Ero1 and Pdi1 in a 1:1 ratio (Fig. 1, lane 1). N-ethylmaleimide (NEM) trapping and nonreducing SDS-PAGE allowed partial trapping of a disulfide linked heterodimer (Fig. 1, lane 1), while expression of the C136A mutant of Ero1 abolished complex formation and copurification (Fig. 1, lane 2). In contrast, coexpression of Erp41 and Ero1 (Fig. 1, lane 5) or Erp41, Ero1, and human PDI (Fig. 1, lane 6) resulted in no copurification of Erp41 with Ero1 (Fig. 1, lanes 5 and 6) and the purified Ero1 was in multiple, mainly monomeric, redox states. Coexpression of *K. phaffii* Pdi1, Ero1, and Erp41 (Fig. 1, lane 7) resulted in the formation of one main redox state for Ero1 and copurification of a minor amount of Pdi1, but not of Erp41. These results confirm that *K. phaffii* Pdi1 is necessary to deliver a correctly folded Ero1 and that it cannot be replaced by Erp41 in the complex.

**Figure 2:**
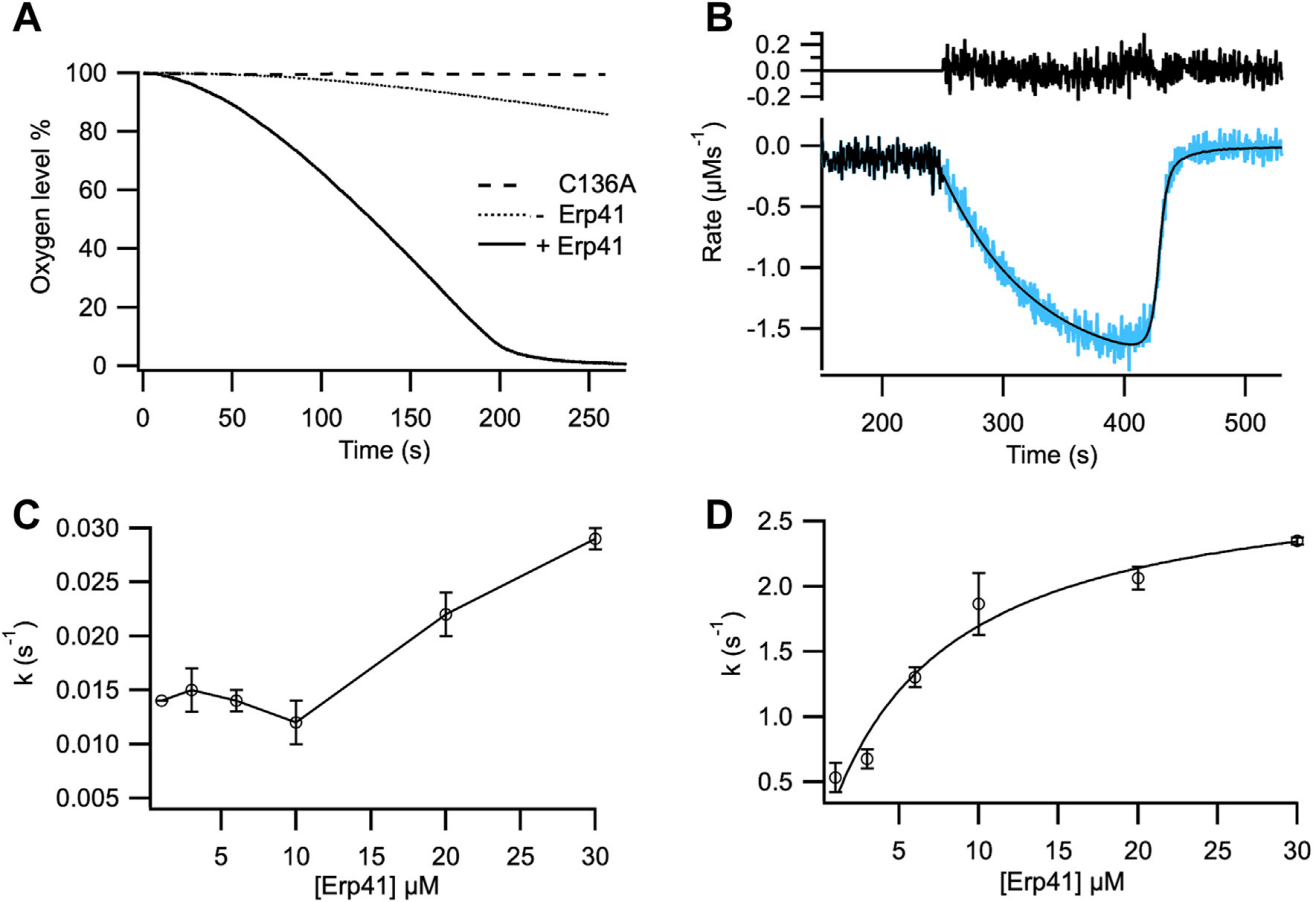
**Oxygen consumption kinetic profiles for Erp41 with Ero1–Pdi1 complex.***A*, representative oxygen consumption trace of *K. phaffii* WT Ero1–Pdi1 complex in combination with 10-fold exogenous *Komagataella phaffii* Erp41 (*solid line*); control reaction with monomeric Ero1^C136A^ without supplied Pdi1 (*dashed line*); reaction catalyzed by Ero1–Pdi1 complex without monomeric Pdi1 or Erp41 (*dotted line*). *B*, differentiated oxygen consumption trace of 1 μM WT Ero1–Pdi1 complex and 1 μM Erp41 fitted to a single step activation model. Residuals are shown above the fitted trace. *C*, titration effect of supplied Erp41 on activation rate. *D*, titration effect of supplied Erp41 on k_cat_, data was fitted to the classical Michaelis–Menten model. Data is shown as mean ± SD (n = 3–5). PDI, protein disulfide isomerase.

We then examined whether Erp41 could act as a catalytic partner for the Ero1–Pdi1 complex. For this, we adopted an assay based on oxygen consumption, as described previously (26, 31), where a PDI family member oxidizes GSH to GSSG and then transfers two reducing equivalents to Ero1, which in turn reduces O_2_ to H_2_O_2_. With no added PDI family member, the rate of reaction of the Ero1–Pdi1 complex is low (Fig. 2*A*), though higher than that of monomeric Ero1^C136A^, possibly due to partial dissociation of Pdi1 from the complex and then its ability to be a substrate for the remaining complex. Addition of Erp41 to a solution containing the complex greatly increased oxygen consumption (Fig. 2*A*), confirming that it acts as an oxidase in partnership with *K. phaffii* Ero1. To obtain kinetic parameters, the derivative of the oxygen consumption trace was fitted to the steady-state Michaelis–Menten model modified for required activation steps and with a Hill coefficient for oxygen (Fig. 2*B*). We previously found that activation of the complex by *K. phaffii* Pdi1 showed the presence of two sequential steps of activation: the first initiated by the neighboring Cys407 of Pdi1 in the complex on the intermolecular disulfide to release Ero1, and the second dependent on monomeric Pdi1, which reduces the Cys76-Cys332 regulatory disulfide on Ero1 (26). As such the first step is independent of added [Pdi1], while the second is dependent. In contrast, with Erp41, we observed just one slow activation step. This suggests that either the mechanism for activation is different with Erp41, or that one activation step is too fast to monitor, or that the kinetics of the two steps are indistinguishable in this experimental set up. A titration of [Erp41] over 1 μM inactive Ero1–Pdi1 complex revealed complex activation kinetics (Fig. 2*C*), with dependence on [Erp41] (equivalent to the second activation step by Pdi1) only at high concentrations (>10 μM). Activation kinetics may be complicated by activation of remaining complexes by Pdi1 released from prior activation reactions. k_cat_ showed a Michaels–Menten dependence on [Erp41] as a substrate for the complex (Fig. 2*D*), with a K_M_ for Erp41 of 7.1 μM, similar to that for Pdi1 (Table 1). At 10-fold excess of Erp41 over the Ero1–Pdi1 complex, the turnover was 1.95 s^−1^, implying Erp41 can recognize and catalyze GSH oxidation in partnership with Ero1 even more efficiently than Pdi1 (k_cat_= 1.28 s^−1^) (Table 1).

**Table 1:** Kinetic parameters derived from oxygen consumption kinetics

Enzymes	Activation t_1/2_ (s)	k_cat_ (s^−1^)	K_M O2_ (μM)	K_M PDI_ (μM)	Hill coeff.
Ero1-Pdi1 + Erp41	58.96 ± 9.04	1.95 ± 0.07	10.81 ± 4.57	7.07 ± 1.17	2.22 ± 0.09
Ero1-Pdi1 + Pdi1 (26)	32.33 ± 1.15	1.28 ± 0.06	13.65 ± 1.81	5.67 ± 2.36	2.36 ± 0.31

Values are derived from 1 μM *Komagataella phaffii* Ero1–Pdi1 complex plus 10 μM Erp41 or Pdi1 or a titration of Erp41/Pdi1 to determine K_M__PDI_. All data is represented as mean ± SD (n = 3–5).

PDI, protein disulfide isomerase.

### Erp41 activity is immune to inhibitory regulation by unfolded proteins and peptides

As Erp41 acts as a catalytic partner to *K. phaffii* Ero1, we then tested the effect of unfolded substrates in the assay. Such intermediates can accumulate during folding stress in the ER and trigger the unfolded protein response (32, 33). Therefore, we employed either reduced bovine trypsin inhibitor (BPTI) as a disulfide-containing model protein or the peptide KFWWFS, which is free of disulfides and simulates the hydrophobic core of unfolded proteins. Addition of either of these results in substrate inhibition of human PDI in its ability to interact with human Ero1 (34, 35). In contrast, *K. phaffii* Pdi1 is immune to this inhibitory regulation, while still being able to refold BPTI and weakly binding the KFWWFS peptide (26). Therefore, we wondered how Erp41 would perform in such conditions, given its uniqueness as a member of the PDI family.

The same assay format as previously (26, 36) was used. The peptide was mixed with Erp41 at the start of the assay to be able to evaluate how its presence might affect both activation phase and catalytic turnover. In contrast, BPTI was added in its reduced state after the Ero1 was fully activated (at 50% residual oxygen) as preincubation with Erp41 could lead to mixed redox states of BPTI, which could hinder the interpretation of the oxygen consumption profiles. Neither generated any measurable effect on the kinetics of activation (KFWWFS only) nor turnover (both substrates) (Fig. S6*A*). This implies that either (i) Erp41 binds neither substrate or (ii) that the interaction of Erp41 with the Ero1–Pdi1 complex is *via* a different mechanism than its interactions with unfolded proteins. Isothermal calorimetry subsequently confirmed lack of binding of Erp41 to the KFWWFS peptide (Fig. S6*B*).

### Erp41 can rapidly oxidize BPTI using glutathione

**Figure 3:**
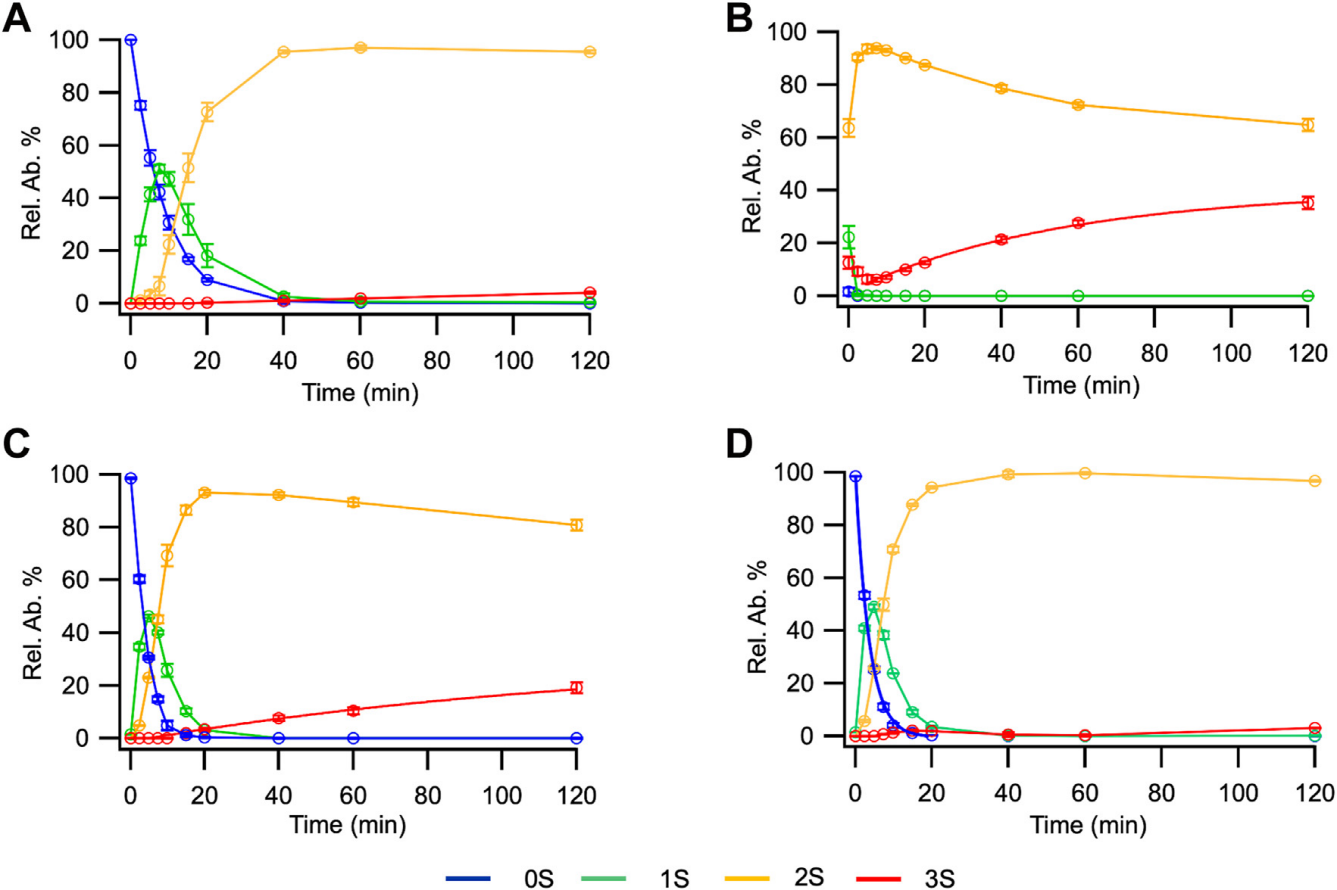
**Erp41-mediated BPTI refolding profiles.***A*, uncatalyzed reaction. *B*, reaction catalyzed by 7 μM Erp41. *C*, reaction catalyzed by 0.1 μM hPDI. *D*, reaction catalyzed by 0.04 μM Erp41. 50 μM BPTI was used in all conditions. n = 3, data represented as average and SD. BPTI, bovine trypsin inhibitor; hPDI, human PDIA1.

Next, we examined whether Erp41 could bind to BPTI and act as a catalyst of disulfide bond formation and isomerization by employing a classical mass spectrometry (MS)-based refolding assay (37). Slow spontaneous oxidation of BPTI is observed in a glutathione redox buffer, resulting in the loss of species with no disulfides (0S), the formation of one disulfide (1S) and two disulfide (2S) intermediates and the final slow formation of the native three disulfide state (3S) (Fig. 3*A*). Often these assays are performed at 0.1 to 7 μM of PDI family member. As Erp41 showed no inhibition by BPTI in the oxygen consumption assay and it lacks the *b’* domain involved in non-native protein binding (38), we expected the kinetics of reaction to be slow and hence we used 7 μM of enzyme. Unexpectedly, the reaction was so fast that all of the 0S and 1S species had disappeared by the first measurement time point (Fig. 3*B*). However, while 3S formation was faster than for the noncatalyzed control reaction, it was still slow and appeared to be plateauing around 40% of 3S. This is in stark contrast to *K. phaffii* Pdi1 (26) or human PDI (Fig. 3*C*), suggesting that Erp41 is an inefficient isomerase. Reducing the concentration of Erp41 from 7 μM to 0.04 μM allowed the formation of 1S and 2S intermediates to be observed (Fig. 3*D*). By fitting to a single exponential function, the rate of oxidation to the 1S state was calculated to be 0.282 ± 0.007 min^−1^ c.f. 0.118 ± 0.001 min^−1^ for the noncatalyzed rate. Once corrected for the non-catalyzed rate this is 3.7-fold faster than for hPDI (Fig. 3*C* and 1. 7-fold faster than for *K. phaffii* Pdi1 (26). Under these biophysical conditions, this makes *K. phaffii* Erp41 the fastest catalyst of disulfide formation using a glutathione buffer that we are aware of. While Erp41 appeared to be an efficient catalyst of disulfide formation, the amount of native species (3S) after 2 h was very low, consistent with the observation at higher [Erp41] that it is an inefficient catalyst of disulfide bond isomerization. This is consistent with its lack of a *b’* domain, which has been shown for hPDI to be required for efficient catalysis of isomerization (39).

**Figure 4:**
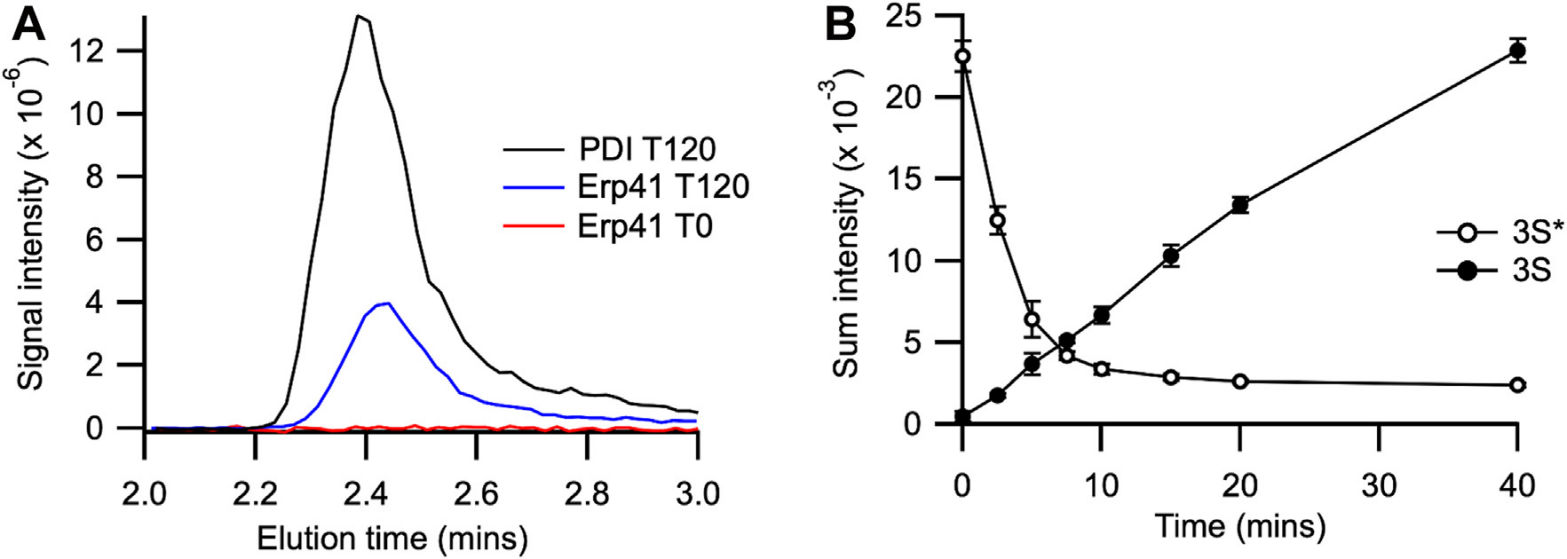
**Examination of native and non-native 3S species of BPTI from refolding with Erp41 at 7 μM.***A*, elution profile at the native 3S BPTI position for BPTI folded by Erp41 for 120 min (*blue*) and immediately after mixing (*red*). For comparison, the 3S elution profile of BPTI refolded for 120 min by PDI (*black*) is shown. *B*, kinetic profile of appearance and disappearance of the non-native 3S∗ species and native 3S BPTI species from Erp41 refolding. Quantification is based on the sum of peak intensities as a function of time, in the form of average and standard error (n = 3). BPTI, bovine trypsin inhibitor; PDI, protein disulfide isomerase.

Further examination of the results for the 7 μM Erp41 catalyzed reaction revealed a phenomenon that we have neither seen nor seen reported before: the 3S species could be detected already present at the first time point (0.2 min) at an abundance of approximately 12.5% (Fig. 3*B*), it then decreased at later time points before subsequently increasing again. This suggests that the early 3S species is non-native and that it is progressively replaced by the native 3S species *via* a 2S species. Re-examination of the reverse-phase HPLC traces from the MS revealed that there was no clear peak for these early 3S species but they eluted around the same region as the ensemble of 2S species and not at the point at which the native protein eluted (Fig. 4*A*). By quantifying the abundance of these two species through the sum of their peak intensities at different elution volumes, we could examine the kinetics of their appearance and disappearance. The non-native 3S (3S∗) can be already detected at the first sampling point (time 0 at 0.2 min) of the reaction and progressively decreases as the native 3S is formed (Fig. 4*B*). This suggests that this represents a mixture of non-native 3S species that may be formed due to the highly efficient catalysis of disulfide formation by Erp41 in a glutathione buffer, but inefficient disulfide isomerase activity of the enzyme.

### The *a’* domain has faster, [GSSG]-independent, peptide oxidation activity

**Figure 5:**
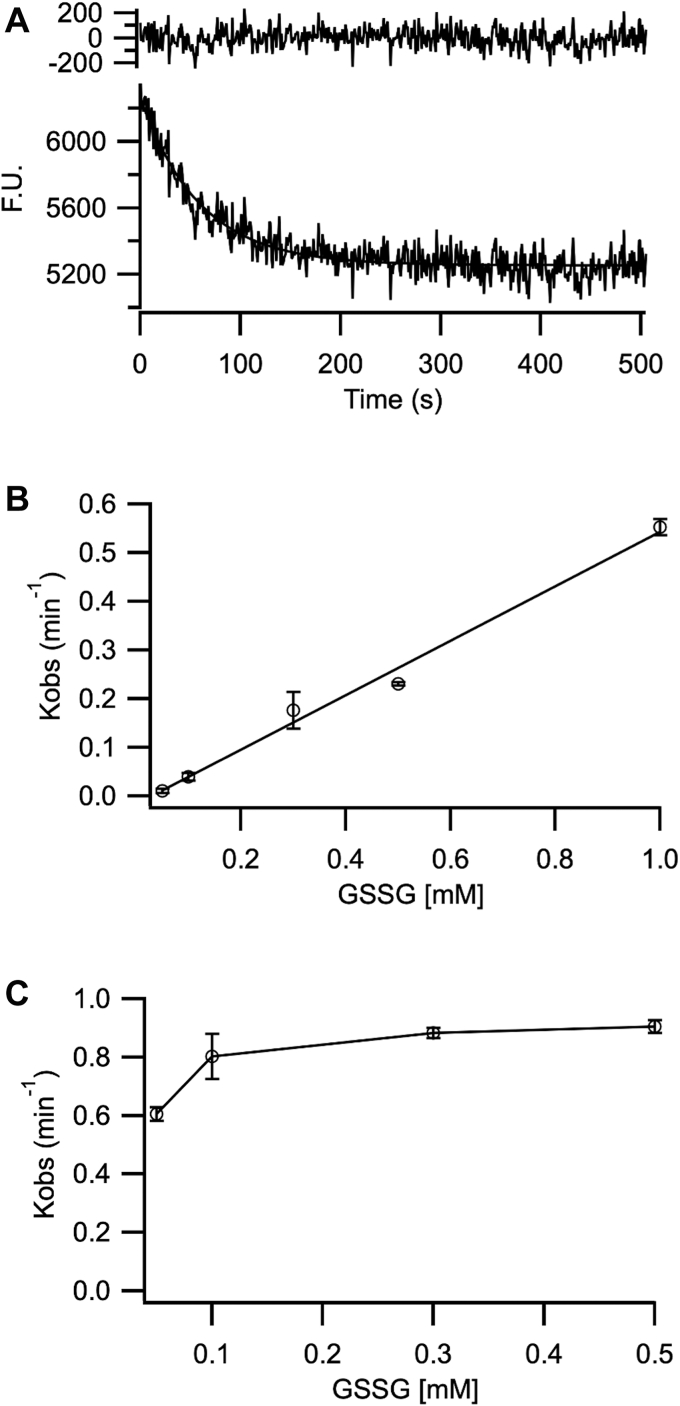
**Peptide oxidation catalyzed by the *a* and *a’* domain of Erp41.***A*, example of fluorescence profile (F.U., fluorescence units) in a peptide oxidation reaction, catalyzed by the *a’* domain in the presence of 0.5 mM GSSG. Residual of the fit to a single exponential function is shown above the trace. *B* and *C*, GSSG titration of the rate of oxidation catalyzed by the *a* domain (*B*) and *a’* domain (*C*). n = 3 to 5, data represented as average and SD.

We hypothesized the high oxidation efficiency observed with BPTI might be related to the presence of the Grx-like active site in the *a’* domain of Erp41 and its high affinity for glutathione. Therefore, we decided to examine the ability of each isolated domain to catalyze glutathione-mediated oxidation. After expressing and purifying the isolated *a* and *a’* domains, we confirmed their identity, secondary structure and stability (Fig. S7, Table S2). We then used a fluorescent peptide-based assay (34) to examine the relative activity and dependence on [GSSG] of each domain. In this assay, the fluorescence of a tryptophan residue in the peptide is monitored, with the fluorescence being quenched as the disulfide bond is formed (Fig. 5*A*). The reaction rates for the *a* domain increased linearly with increasing [GSSG] (Fig. 5*B*), indicating that the rate limiting step is reoxidation of the active site by GSSG. In contrast, the reaction rates for the *a’* domain remained constant at around 0.8 min^−1^, except at the lowest concentration of GSSG tested (Fig. 5*C*), implying that over this [GSSG] range, oxidation of the peptide is rate-limiting. This suggests that the rate of oxidation of the Grx-like active site of the *a’* domain by GSSG is much faster than that of the *a* domain due to its higher affinity for glutathione.

### Glutathione-mediated oxidoreduction of the Erp41 *a’* domain active site is rapid

To investigate the glutathione-mediated oxidoreduction of the *a’* domain in more detail we performed stopped-flow kinetics, as described in previous studies for the *a* domain of human and *S. cerevisiae* PDI (40). The quenching of W^151^, which immediately precedes the CGYC active site motif, was used as a spectroscopic marker to monitor both oxidation and reduction. Quenching of W^151^ is caused by adjacent disulfide formation in the active site. As in previous studies of hPDI (36, 37), this necessitated mutation of the second tryptophan present in the domain (W^162^ in Erp41) into phenylalanine, as the fluorescence of W^162^ would otherwise mask the fluorescence signal of W^151^. Biophysical characterization was performed to ensure that no significant effects on the protein structure arose from the W151F mutation (Fig. S8).

**Figure 6:**
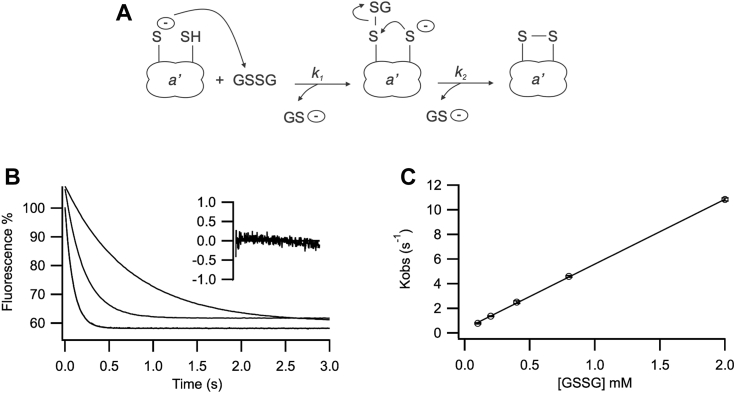
**Stopped-flow kinetics of oxidation of the *a’* domain by GSSG.***A*, mechanism of GSSG-mediated dithiol oxidation. *B*, representative traces of the kinetics of oxidation of the *a’* domain of Erp41^W162F^ in the presence of 0.2 mM, 0.8 mM, and 2 mM GSSG. Residuals are shown for one fit to a single exponential function at 0.2 mM GSSG. *C*, linear regression of the k_obs_ values on [GSSG]. Data is shown as mean ± SD (n = 4–7).

GSSG-mediated oxidation of the active site is a two-step process (Fig. 6*A*). In the first step, the N-terminal active site cysteine undergoes a nucleophilic attack on GSSG, forming a mixed disulfide. This is a second order reaction. When GSSG is in large excess over the protein substrate (as performed here), this simplifies to a pseudo first order reaction, yielding rate constants which are proportional to [GSSG]. In the second step, the C-terminal active site cysteine resolves the complex, resulting in formation of an intramolecular disulfide in the active site. This step is GSSG-independent. Upon oxidation of the *a’* domain of Erp41 the fluorescence decreases as a consequence of W^151^ quenching. The kinetic profile shows random residuals when fitted to a single exponential function (Fig. 6*B*). Therefore, only one of the two expected rates could be detected. This rate constant showed a linear dependence on [GSSG] (Fig. 6*C*) implying it corresponds to the first reaction step, with a derived second order rate constant of 5265 ± 45 M^−1^ s^−1^. This is approximately 30-fold faster than the rate constant for the *a* domain of hPDI (191 ± 3 M^−1^ s^−1^ (40)) or *S. cerevisiae* PDI (163 ± 5 M^−1^ s^−1^ (40)) for the formation of the mixed disulfide intermediate. The second rate constant could not be observed, despite MS data confirming that the reaction went to completion. This could arise either due to no spectroscopic change being present in the conversion of the mixed disulfide to the intramolecular disulfide state or due to this step being too fast to be measured.

**Figure 7:**
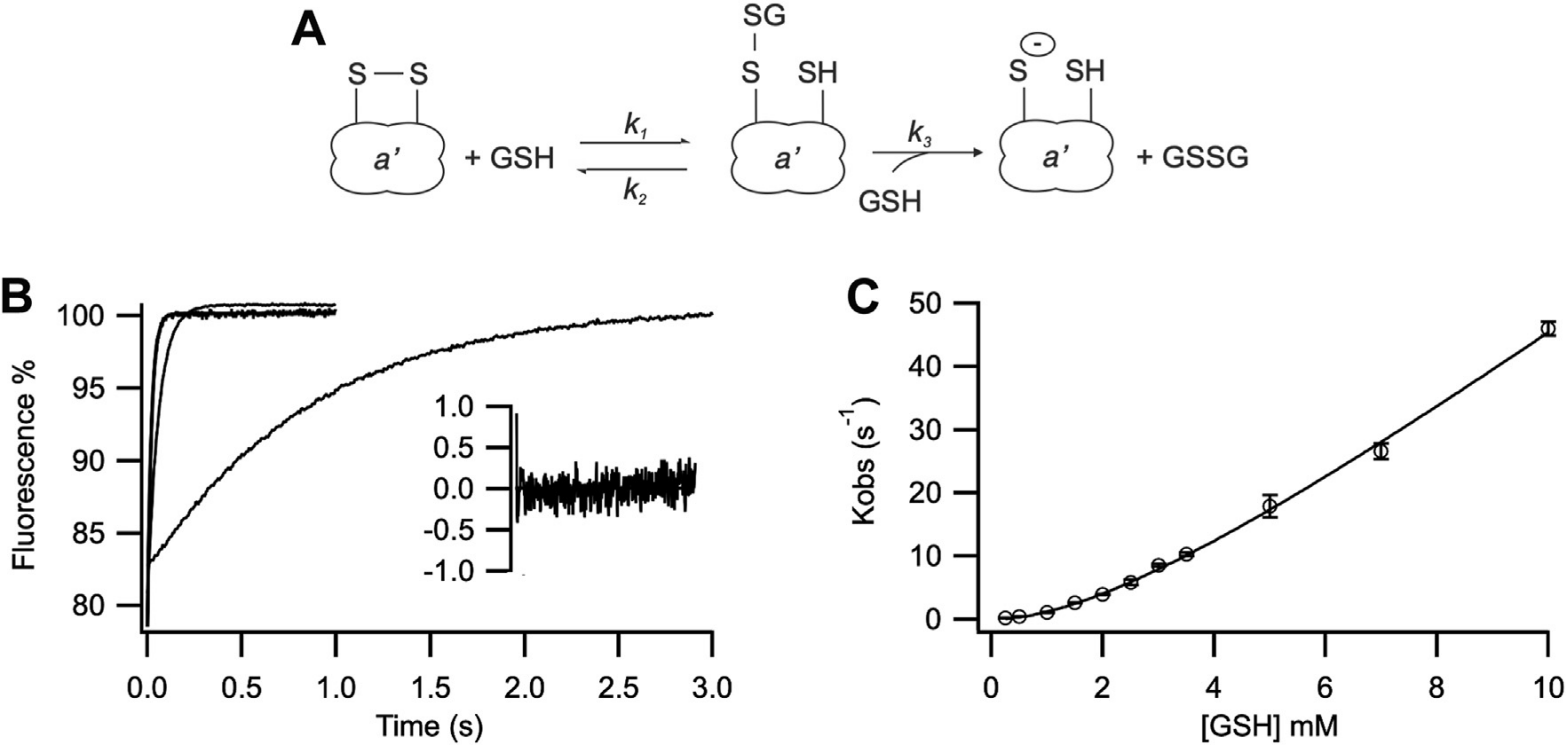
**Stopped-flow kinetics of reduction of the *a’* domain active site by GSH.***A*, mechanism of GSH-mediated reduction including kinetic partitioning. *B*, representative traces of the kinetics of reduction of the a’ domain (W162F) in the presence of 1 mM, 5 mM, and 10 mM GSH. Residuals are shown for one fit to a single exponential function at 1 mM GSH. *C*, nonlinear regression of the k_obs_ values on [GSH]. Data is shown as mean ± SD (n = 4–7).

The reduction of the active site by GSH is also a two-step process. Both reactions are GSH-dependent and therefore second order but with GSH in large molar excess both simplify to pseudo first order reactions. The reduction can also undergo kinetic partitioning: if the GSH concentration is low enough, the C-terminal cysteine of the mixed disulfide complex competes with exogenous GSH, bringing the complex back to the initial oxidized state (Fig. 7*A*). The fluorescence profile during GSH-mediated reduction fitted to a single-exponential process (Fig. 7*B*). A plot of K_obs_ values with increasing GSH concentrations showed linearity when [GSH] >2 mM (Fig. 7*C*). At lower concentrations of GSH, linearity is lost because of the partitioning with the reverse reaction taking place. By fitting how K_obs_ varies with [GSH], we obtained values for the three rate constants of: k_1_ = 6560 M^−1^ s^−1^, k_2_ = 70.4 s^−1^, and k_3_ = 15,800 M^−1^ s^−1^. Both forward rates are higher than those reported for the *a* domain of hPDI (k_1 hPDI_ = 4600 M^−1^ s^−1^, k_3 hPDI_ = 11,200 M^−1^ s^−1^), while the reverse rate is approximately 6.1-fold slower (k_2 hPDI_ = 430 s^−1^) (40), which is consistent with the Grx-like active site stabilizing the mixed disulfide state. The k_3_/k_2_ ratio is 225 for the *a’* domain of Erp41, which is 8.6× higher than that of the *a* domain of hPDI and 1.9× higher than that of the *a* domain of *S. cerevisiae* Pdi1 (40). This means that reduction of the active site by GSH is more efficient at lower [GSH] for Erp41 *a‘* domain. This is comparable to the more efficient oxidation rates at lower [GSSG] seen above. This suggests that the reactivity with GSH of the *a’* domain of Erp41 is fundamentally different from that of the *a* domain of human or *S.cerevisiae* PDI *a* domain.

## Discussion

*K. phaffii* and *Yarrowia* spp. are nonconventional yeasts used in a variety of industrial applications. *K. phaffii* is a methylotroph and a common host for protein production capable of generating high titers of industrial target proteins (25, 41, 42), while the genus *Yarrowia* gathers oleaginous yeasts, among which *Y. lipolytica* is the most common for industrial applications (43, 44). So far, only one study has been published, which focus on biochemical aspects of oxidative folding pathways in *K. phaffii* (26), and it largely remains unstudied in *Yarrowia*. As for all eukaryotic organisms, disulfide bond formation in *K. phaffii* is carried out in the ER by members of the PDI family and Ero1. *K. phaffii* has four PDI paralogs: Pdi1, Mpd1, Eps1, which are found also in the model yeast *S. cerevisiae*, and Erp41, which is not present in *S. cerevisiae*. A homolog of Erp41 is also predicted to be encoded in *Y. lipolytica* and a small number of other species (Table S1).

Erp41 has two thioredoxin-like domains: *a* and *a’.* Proteins with a similar domain architecture are also present in different ascomycete yeast and filamentous fungi, however, Erp41 differs from them, as the active site in the *a’* domain includes a tyrosine in the motif CXYC. The presence of Y/F residues at this position between the catalytic cysteines is a characteristic trait of Grxs and is known to increase affinity for glutathione (12, 45). We are not aware of prior studies on a PDI family member combining classical PDI-like and Grx-like active sites in a single protein. Erp41 has some properties akin to those of Pdi1, but also many differences. Both are able to function with *K. phaffii* Ero1 in catalyzing disulfide bonds, with Erp41 showing a faster turnover, but slower activation kinetics than Pdi1. Interestingly, while Pdi1 forms an inactive complex with Ero1 and is able to promote folding of Ero1 to a single redox state, Erp41 cannot do either. This implies that the mechanisms of Ero1 binding and recognition by Erp41 must be fundamentally different from that of Pdi1. The biggest difference between Erp41 and Pdi1 lies in their interaction with glutathione, both GSSG and GSH. In fact, Erp41 is able to catalyze rapid oxidation of peptide and protein substrates, with this being limited by peptide oxidation rather than catalyst reoxidation by GSSG. For BPTI, this oxidation is so fast that non-native 3S BPTI species can be observed under some conditions. In contrast, Erp41 is inefficient at catalyzing disulfide isomerization, possibly due to it lacking a substrate binding *b’*-like domain, something previously shown to be required for catalysis of isomerization by hPDI (39).

We hypothesized that the rapid kinetics of oxidation in a glutathione-based buffer was due to the Grx-like active site of the *a’* domain. Examination of the GSSG dependence of peptide oxidation by the *a* and *a’* domains and examination of the rates of reaction of both actives sites with GSH/GSSG revealed this to be true. While the *a* domain behaved like a classical PDI, the *a’* domain showed very rapid kinetics of reaction with glutathione. Dissection of individual rates by stopped-flow revealed that the rate of reaction of the *a’* domain of Erp41 was circa 30-fold faster with GSSG and circa 60-fold faster with GSH than that previously reported for the *a* domain of *S. cerevisiae* Pdi1.

Altogether, these results indicate that Erp41 is a special member of the PDI family, which can oxidize disulfides with high efficiency when in partnership with glutathione and is able to retain this efficiency even at low glutathione concentrations. While Erp41 is not essential, the rapid *in vitro* kinetics suggests that the Erp41/glutathione axis may form a major physiological route for disulfide formation in *K. phaffii.* In addition, it further augments one of the conclusions drawn from our prior analysis of the *K. phaffii* Ero1–Pdi1 axis (26): it is problematic to extrapolate kinetic parameters and pathways for oxidative folding between species, rendering the assumption of equivalence as potentially inexact.

## Experimental procedures

### Vector assembly

Bacterial expression vectors were generated with classical restriction digestion between NdeI and BamHI. Protein coding genes were ordered from Twist Bioscience and codon optimized for *E. coli,* while restriction sites were added by PCR. A pET23 backbone with a lactose inducible Tac promoter was used for the expression of all His-tagged proteins (46). Single nucleotide variants were obtained with site-directed mutagenesis protocols (QuickChange mutagenesis kit, Stratagene). *E. coli* XL1-blue (Agilent) was used as a propagation strain for all bacterial constructs. Yeast vectors were generated using Golden Gate cloning in the GoldenPiCS library adapted for *K. phaffi* (47). The list of constructs used in this study is reported in Table S2.

### Erp41 KO generation

The Erp41 knock out was generated following the protocol described in Gassler *et al.* (48), for CRISPR homology-directed gene targeting in *K. phaffii*. Briefly, *K. phaffii* CBS7435 cells were transformed by electroporation with a plasmid expressing the SpCas9 gene under pLAT1 and the sgRNA under pGAP and a linear repair template. pLAT1 and pGAP are, respectively, a moderate and a strong promoter in the presence of glucose as a carbon source. To increase efficiency, two different guides targeting the 5′ of the gene were designed, one annealing to the forward strand and the other to the reverse strand. Two constructs were assembled with either nourseothricin or G418 resistances, each carrying one guide, and transformed together. The homology regions to generate the repair template were selected 500 bp upstream and downstream of the gene, connected with a BsaI site, cloned into the Golden Gate vector system and from there amplified to obtain a linear 1000 bp fragment. Approximately 100 ng of each plasmid and 3000 μg of repair template were combined in the transformation mix. Transformants growing on nourseothricin and G418 were screened with colony PCR, both with primers annealing outside of the targeted locus and of the region used for homology-based repair, and with gene-specific primers, in order to exclude reintegration events (as shown in Fig. S2). The list of oligonucleotides used in this study is reported in Table S3.

### Viability assessment

Erp41 KO clones were tested for viability on solid and liquid media, respectively, through spotting assays and growth experiments with real time *A*_600_ monitoring in a Sunrise photometer (Tecan AG). Liquid precultures were carried out in 24-deepwell plates, in 2 ml yeast extract peptone dextran (YPD) media at 25 °C and 250 rpm shaking.

For spotting assays, *A*_600_ was measured after 24 h, cells were washed in 20 mM sodium phosphate, 150 mM NaCl, pH 7, and growth was leveled to *A*_600_ 0.3 for all cultures. Serial factor ten dilutions were made and 5 μl were spotted on YPD and yeast nitrogen base plates each with 2% glucose, either plain or supplemented with tunicamycin, diamide, or DTT in different concentrations. Plates were incubated at 30 °C and growth was monitored daily. Spotting plates displayed one WT control and four biological replicates of the Erp41 knock out. Three to five replicates were performed for each condition.

For growth in liquid media, preculture *A*_600_ was measured after 24 h, cells were washed in YPD with and without supplemented 2.5 μM tunicamycin and leveled to *A*_600_ 0.12. A volume of 180 μl was aliquoted in the wells of a transparent 96-well plate for each culture. The outermost rectangle was filled with 200 μl water to prevent evaporation. A total of four biological replicates were tested, together with the WT, each assayed in five technical replicates. The plate was incubated in the Sunrise photometer at 30 °C, 250 rpm shaking, and *A*_600_ monitoring every 15 min for 72 h.

### Protein expression and purification

For small-scale coexpressions, *E. coli* main cultures were grown in a 24-deepwell plate format in 2 ml autoinduction medium (Formedium) + 0.8% glycerol at 250 rpm shaking, at 20 °C for Erp41, Pdi1, Ero1-Pdi1 complex, Ero1^C136A^, Erp41-Ero1, and Erp41-Ero1 with hPDI or *K. phaffii* Pdi1 CyDisCo (26, 49, 50). For large-scale productions, full-length Erp41 was produced in *E. coli* HMS174(DE3) and grown in 2.5 L UltraHigh yield flasks (10% fill volume), in autoinduction medium (Formedium) + 0.8% glycerol with 250 rpm shaking at 15 °C, supplemented with antifoam 204 (Sigma-Aldrich). Isolated Erp41 domains (a, a’ WT and W162F) were produced in *E. coli* BL21 *ΔTrxAΔTrxC* and grown in several 250 ml flasks (10% fill volume) with 250 rpm shaking at 15 °C. Preculture inoculum was kept to 1/100 of the main culture volume for small- and large-scale expressions. 100 μg/ml ampicillin (for the target protein expression plasmid) and/or 35 μg/ml chloramphenicol (for CyDisCo maintenance) were supplemented at all stages. Cultures were harvested at 48 h from inoculation in the autoinduction media. Small-scale pellets were resuspended to the initial volume with 20 mM sodium phosphate, 150 mM NaCl, pH 7, mixed with 100 μg/L lysozyme and 20 μg/ml DNaseI, incubated at 30 °C for 15 min, lysed by freeze-thawing, and cleared by centrifugation.

Purification was carried out on Poly-Prep columns (Bio-Rad) with 0.4 ml bed volume of HisPur Cobalt Agarose resin (Thermo Fischer Scientific) and 1 ml lysate, as described by (50), with a final elution step in 50 mM EDTA in 20 mM sodium phosphate, 150 mM NaCl, pH 7. Pellets from large-scale expressions were resuspended to the original culture volume in a buffer containing 20 mM sodium phosphate, 150 mM NaCl, and 20 μg/ml DNaseI, lysed by sonication, cleared by centrifugation, and loaded on a pre-equilibrated 5 ml HisTrap HP column (Cytiva). Elution was carried out in ten column volume imidazole gradient from 0 to 300 mM in 20 mM sodium phosphate, 150 mM NaCl, pH 7. Eluate was quickly buffer exchanged in 20 mM sodium phosphate, 150 mM NaCl, pH 7, and filtered on a pre-equilibrated Superdex 200 16/600 HiLoad column (Cytiva). After SDS-PAGE visualization, the purest fractions were pooled, flash-frozen in liquid nitrogen, and stored at −70 °C. Protein concentration was determined with theoretical molecular masses, extinction coefficients, and absorbance measurements at 280 nm (KpErp41 MW = 41,184.6 Da, ε_280_ = 34,630 M^−1^ cm^−1^; KpErp41 *a* domain MW = 14,010.97 Da, ε_280_ = 14,440 M^−1^ cm^−1^; KpErp41 *a’* domain MW = 14,474.1 Da ε_280_ = 16,960 M^−1^ cm^−1^; KpErp41 *a’* domain W/F MW = 14,435.1 Da ε_280_ = 11,460 M^−1^ cm^−1^).

### Intact protein MS

Purified protein samples were diluted to 0.1 mg/ml in 20 mM sodium phosphate pH 7 and either directly acidified with trifluoroacetic acid (final concentration 0.1%) or preincubated for 10 min in 25 mM NEM both in the presence and absence of 4 M guanidium chloride as a denaturant. Samples were separated on a Waters BioResolve TMRP mAB polyphenyl column (450 Å, 2.7 μm, 2.1 × 50 mm) in 0.1 to 0.5% formic acid with increasing acetonitrile gradient and analyzed by electrospray ionization MS on a Q-Exactive Plus Orbitrap instrument (Thermo Fisher Scientific).

### Circular dichroism

CD spectra were recorded on a Chirascan-plus CD spectrophotometer (Applied Photophysics) between 280 and 185 nm at 22 °C in 0.1 cm optical path quartz cuvette. CD measurements were acquired every 1 nm with 0.5 s integration time and repeated three times with baseline correction. Samples were measured in 10 mM sodium phosphate pH 7. Data were processed using Chirascan Pro-Data Viewer (Applied Photophysics).

### Thermostability

Thermostability curves were recorded in four replicates per sample on a Thermofluor CFX96 thermocycler.

Purified protein samples were diluted to 0.5 mg/ml in 20 mM sodium phosphate, 150 mM NaCl, pH 7, mixed with SYPRO Orange protein stain (Thermo Fischer Scientific). Denaturation cycle was carried out between 10 °C and 95 °C in increments of 0.5 °C for 10 s. Triplicate curves were collected for all samples.

### SDS-PAGE and sample preparation

Reduced SDS-PAGE samples were prepared by mixing protein samples with loading buffer containing 100 mM DTT, left incubating for 15 min at room temperature (RT) and heated up at 95 °C for 5 min. Nonreduced samples were treated with NEM at the final concentration of 25 mM for 15 min, mixed with loading buffer without reducing agent and heated up at 95 °C for 5 min. Samples were run on commercial 20% acrylamide gels (Bio-Rad) in a tris/glycine/SDS running buffer (Bio-Rad) for 30 min at 200 mV and the gels scanned on a Bio-Rad Molecular Imager FX after staining with Coomassie blue.

### Oxygen consumption assay

Activity of Ero1 was assessed by oxygen consumption with a Clark-type oxygen electrode (Oxytherm, Hansatech Ltd). Experiments were conducted essentially as described (26). Briefly, 1 μM of Ero1–Pdi1 complex was injected into a reaction mixture containing 2 mM EDTA, 150 mM NaCl, 10 μM Erp41, 50 μM flavin adenine dinucleotide, 10 mM GSH, and 20 mM sodium phosphate at pH 7. To keep the catalytic cycle active until oxygen would become the only limiting substrate in the assay, 1 mM NADPH and 0.05 U/μl glutathione reductase were added to the reaction mix. As a starting point, Erp41 and GSH were incubated in the reaction chamber for 4 min, so that both active sites could be reduced, after which the Ero1–Pdi1 complex was injected. Reaction was recorded until oxygen levels were completely depleted. The control reaction without Erp41 was recorded similarly with injecting 1 μM of Ero1^C136A^ mutant instead of the Ero1–PDI complex. Inhibitors were added at 50 μM either before Ero1 was injected to the assay or at approximately 50% oxygen saturation. Injection volumes of inhibitors were kept to less than 20 μl to avoid the risk of perturbations. Reactions were recorded until complete oxygen depletion was observed. Three to five traces were collected per reaction condition. Kinetic parameters such as k_cat_, activation rates, K_M_, and Hill coefficient were obtained with the fitting functions described in Moilanen *et al.* (31).

### Isothermal calorimetry

Protein–ligand interaction experiments were performed on a MicroCal isothermal calorimetry–200 (Malvern) calorimetry instrument. Erp41 and KFWWFS peptide were diluted in 20 mM sodium phosphate, 150 mM NaCl, pH 7 to the final concentrations of 50 and 750 μM, respectively. The titration was carried out at 25 °C with an initial injection of 0.4 μl, followed by 2.44 μl peptide to protein injections at 3 min intervals. The data was analyzed using OriginLab (OriginLab Corporation). The first data point corresponding to the first injection was excluded from the final set, as advised by the manufacturer.

### BPTI oxidation and refolding assay

Both assays were performed in a reaction buffer containing 0.5 mM GSSG, 2 mM GSH, 0.1 M phosphate, and 1 mM EDTA at pH 7. A similar protocol as in Woehlbier *et al.* (37) was employed: the oxidation reactions were carried out with 50 μM BPTI and when present 0.1 μM hPDI and 7 μM or0.04 μM Erp41. Reactions were sampled at distinct intervals and rapidly quenched with 100 mM NEM, left incubating for 1 min, flash-frozen in liquid nitrogen and stored at −70 °C until analyzed by electrospray ionization MS. All reaction conditions were performed in triplicates.

### Oxidase assay

The method described in Ruddock *et al.* (51) was employed, where a fluorescent decapeptide (NRCSQGSCWN) is used to determine the oxidase activity of the Erp41 domains. The enzyme (0.2 μM) was incubated for 1 min in the reaction mixture in a 315 μl quartz cuvette, containing 0.5 mM GSSG in Mcllvaine buffer pH 7, before adding the substrate peptide (pre-equilibrated to RT) to the final concentration of 5 μM. The reaction was quickly mixed, and the fluorescence decay was monitored in a Fluoromax-4 (Horiba Scientific) fluorometer. All measurements were performed at 25 °C, excitation 280 nm, emission 356 nm, and slit width of 1 nm for excitation and 5 nm for emission. Data was fitted to a single exponential model.

### Stopped-flow kinetics

Oxidation and reduction of the active site of the *a’* W162F domain were determined by the change of fluorescence of the tryptophan adjacent to the N-terminal active site cysteine. Fluorescence was recorded on a KinTek SF-2004 stopped-flow fluorometer, 280 nm excitation, >320 nm bandpass emission at 25 °C, 10 μM enzyme with 0.25 to 10 mM GSH, or 0.1 to 2 mM GSSG, in 20 mM sodium phosphate buffer pH 7 and 2 mM EDTA, as in Lappi *et al.* (40). To ensure complete oxidation or reduction of the active site, the purified domain was incubated with 5-fold excess DTT or GSSG for 30 min at RT, and then exchanged in reaction buffer with 5 kDa Amicon filters (Sigma-Aldrich) at 4 °C. Each data point was averaged using 4 to 7 traces deriving both from the same sample dilution and multiple dilutions performed on different days to ensure solid reproducibility. Data was fitted to a single exponential model.

## Data availability

All data is included in the paper.

## Supporting information

This article contains supporting information.

## Conflict of interests

A patent for the CyDisCo plasmid system is held by the University of Oulu: method for producing natively folded proteins in a prokaryotic host (Patent number 9238817; date of patent January 19th, 2016). Inventor: L. W. R. The other authors declare that they have no conflict of interests with the contents of this article.
