# Supplementary material for: Komagataella phaffii Erp41 is a protein disulfide isomerase with unprecedented disulfide bond catalyzing activity when coupled to glutathione
Source: J Biol Chem. 2024 Feb 13;300(3):105746. doi: 10.1016/j.jbc.2024.105746 (PMC10938136; doi:10.1016/j.jbc.2024.105746)
Supplement: Supporting Tables S1–S4 and Figures S1–S8 [file mmc1.pdf]

Supporting information

***Komagataella phaffii* Erp41 is a protein disulfide isomerase with unprecedented disulfide bond catalyzing activity when coupled to glutathione**

Arianna Palma [1 2], Lukas A Rettenbacher [3 4], Antti Moilanen [4], Mirva Saaranen [4], Brigitte Gasser [1 2], Lloyd W. Ruddock [4*]

[1]Institute of Microbiology and Microbial Biotechnology, Department of Biotechnology, University of Natural Resources and Life Sciences (BOKU), Vienna, Austria.

[2]Austrian Centre of Industrial Biotechnology, Vienna, Austria.

[3]School of Biosciences, University of Kent, Canterbury, UK.

[4]Faculty of Biochemistry and Molecular Medicine, University of Oulu, Oulu, Finland.

* Corresponding author: lloyd.ruddock@oulu.fi

The supporting information consists of eight figures (S1-S8) and four tables (S1-S4)

Figure S1: Domain architecture and AlphaFold model of mature Erp41. *A*. Domain architecture of *K. phaffii* Erp41 according to InterPro conserved domain search; *B*. The *a* and *a'* domains, with their respective catalytic cysteines and neighbouring tryptophans, and the catalytically inactive *c* domain are labelled.

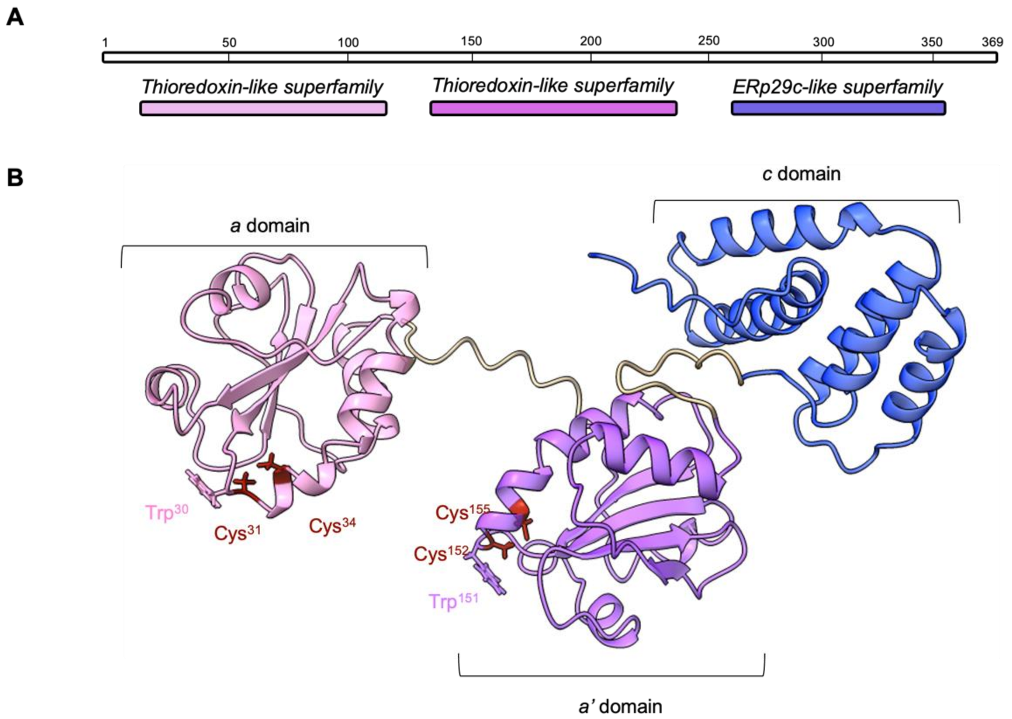

Figure S2: Erp41 knock-out strategy. Schematic representation of the wild-type and knocked-out loci including primers used for PCR-based verification of a successful deletion. Gels for locus PCR and gene specific PCRs are shown below. DNA was stained with Midori Green and the gels scanned in a Gel Doc UV Imaging System (BioRad).

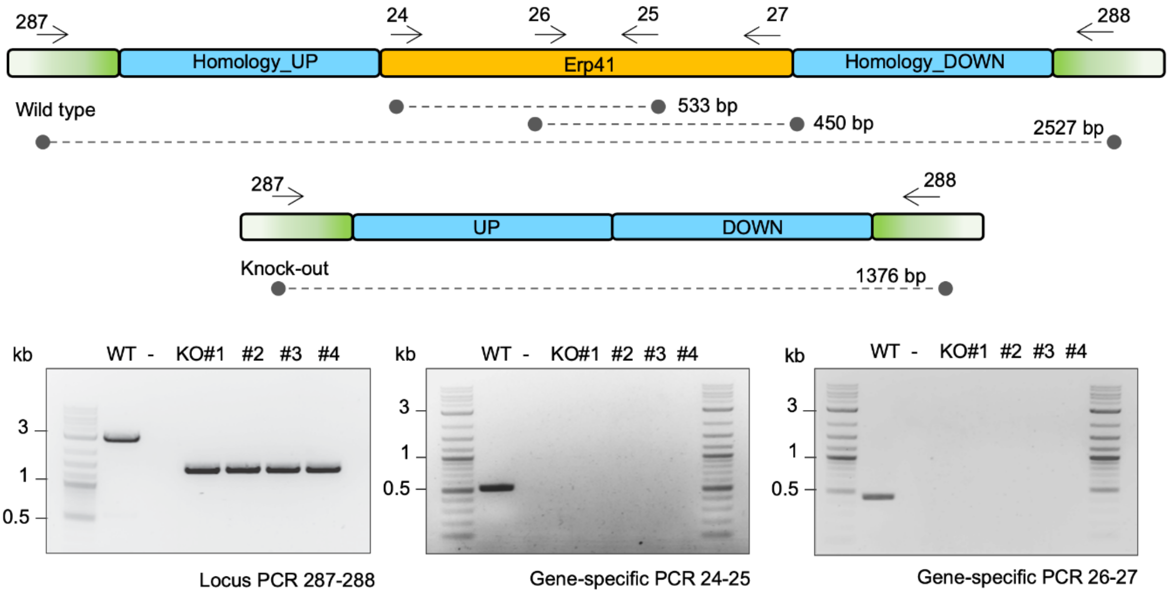

Figure S3: Phenotypical characterization of the Erp41 knock-out strains. *A.* Spotting assays of wild-type (WT) and KO strains (#1-4) performed on YPD and YNB, plain or supplemented with several concentrations of tunicamycin, diamide or DTT. Results obtained with 1 mM diamide, 1.5 μM tunicamycin, 2.5 mM DTT in YNB and 20 mM DTT in YPD were selected for display. *B.* Growth curves of wild-type (WT) and KO strains (#1-4) in liquid YNB, plain and supplemented with 2.5 μM tunicamycin.

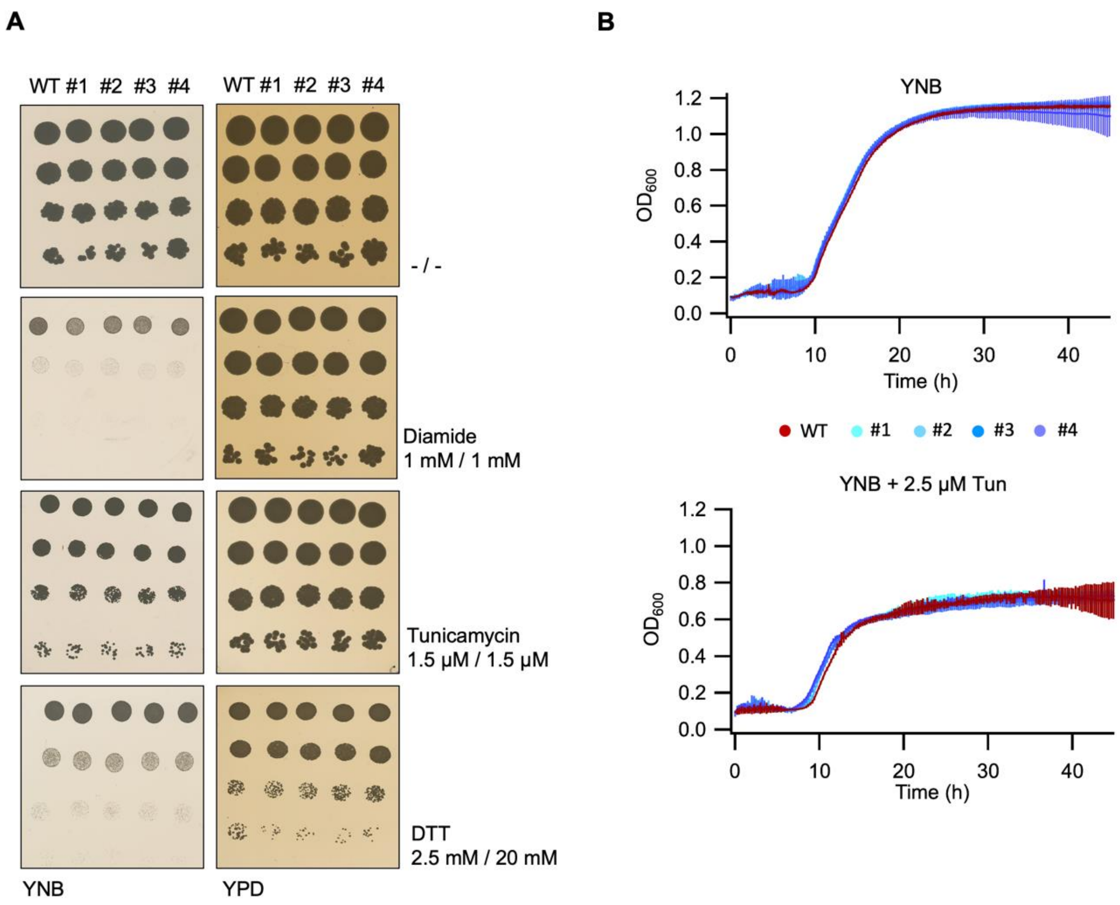

Figure S4: Secondary structure and stability assessment for Erp41 *A.* Mean residue molar ellipticity (MRME) spectra. *B*. RFU derivative curves over temperature.

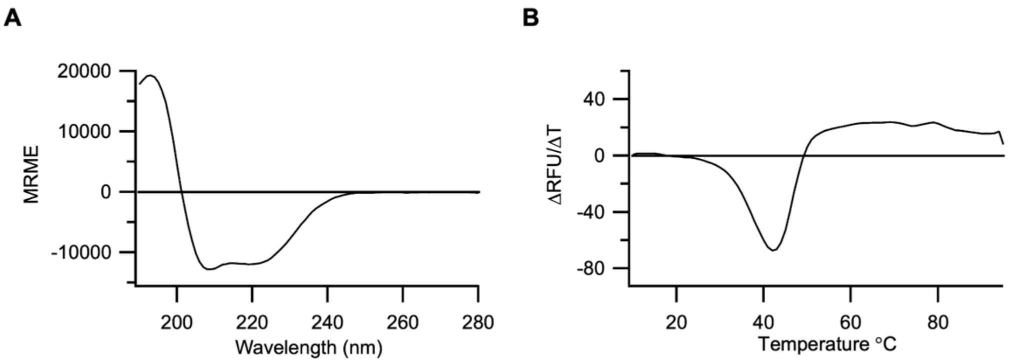

Figure S5: Full uncropped image of SDS-PAGE gel shown in Figure 2.

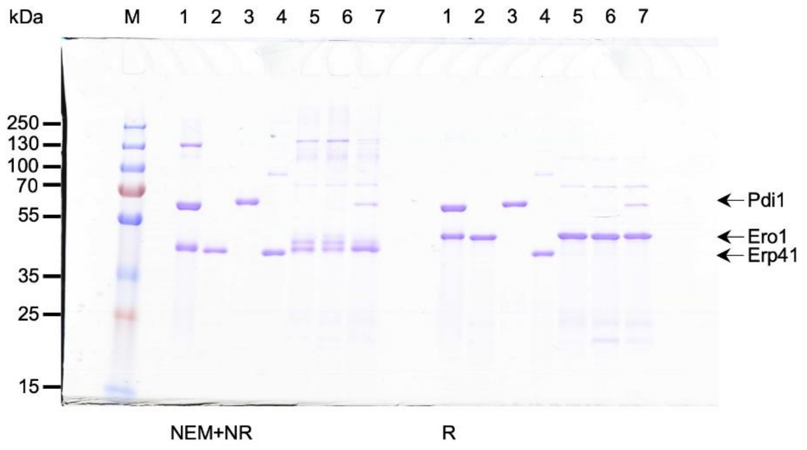

Figure S6. Effects of and affinity for folding inhibitors. *A.* Oxygen consumption traces of the Ero1-Pdi1 complex in the presence (positive control) and absence (negative control) of exogenous Erp41, overlapped with traces including 5-fold excess BPTI (injected at 50% residual oxygen) or 5-fold excess KFWWFS peptide (present from the beginning of the assay). n=3-5, one representative trace is shown; *B.* ITC titration profile of 50 μM *K. phaffii* Erp41 and 0-750 μM KFWWFS peptide.

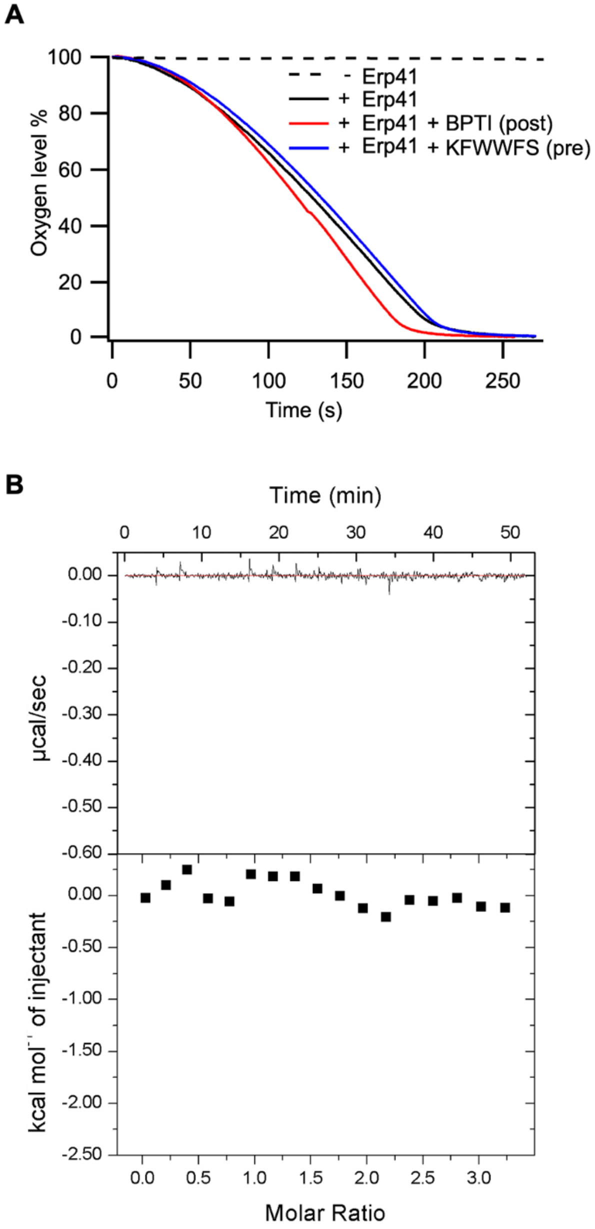

Figure S7: Secondary structure and stability assessment for Erp41 catalytic domains *A.* Mean residue molar ellipticity (MRME) spectra of Erp41 *a* (red) and *a'* (black) domains. *B.* RFU derivative curves over temperature of *a* (red) and *a'* domain (black).

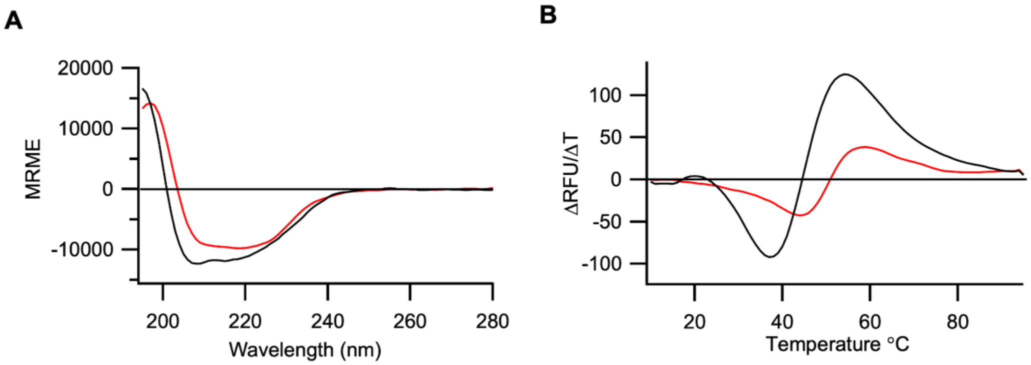

Figure S8: Secondary structure and stability assessment for W/F mutant in Erp41 *a'* domain. *A.* Mean residue molar ellipticity (MRME) spectra of Erp41 *a'* wild-type (continuous line) and *a'* $^{W/F}$ (dashed line) domains. *B.* RFU derivative curves over temperature of *a'* (continuous line) and *a'* $^{W/F}$ domain (dashed line).

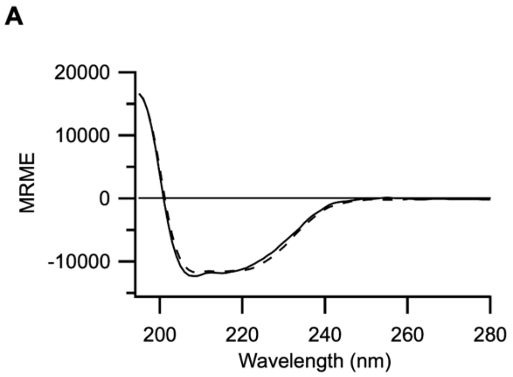

Supplementary Tables

Table S1: Erp41 sequence homology search. The first 6000 BLAST matches to the *K. phaffii* Erp41 protein sequence were filtered for at least 30% sequence identity, 90% query coverage and the presence of 2 CXXC motifs, at least one of which having either GY or the GF between the two cysteines. Sequence variants from the same organism were removed and only the one with the highest Bit score was included.

Sequence_ID	Organism	Description	Active sites	Bit_Score	E_Value	Identity (%)
XP_002489806.1	*Komagataella phaffii*	Methylotrophic yeast	CSHC/CGYC	724.16	0.00E+00	100.00
KAI0465073.1	*Komagataella kurtzmanii*	Methylotrophic yeast	CSHC/CGYC	717.61	0.00E+00	99.15
ANZ74444.1	*Komagataella pastoris*	Methylotrophic yeast	CSHC/CGYC	676.01	0.00E+00	92.07
XP_501758.1	*Yarrowia lipolytica*	Oleaginous yeast	CGHC/CGYC	221.48	3.36E-65	36.08
KAF3294879.1	*Orbilia oligospora*	Nematophagous fungus	CGHC/CGYC	220.71	9.80E-65	37.21
RVD86524.1	*Arthrobotrys flagrans*	Nematophagous fungus	CGHC/CGYC	217.62	2.05E-63	37.03
KAI8870065.1	*Ramicandelaber brevisporus*	Saprotrophic fungus	CGHC/CGYC	204.91	2.12E-58	36.65
XP_056037743.1	*Schizosaccharomyces osmophilus*	Fission yeast	CGHC/CGYC	201.83	1.65E-57	36.39
XP_013020710.1	*Schizosaccharomyces octosporus*	Fission yeast	CGHC/CGYC	199.52	1.07E-56	35.82
EWC48572.1	*Drechslerella stenobrocha*	Nematophagous fungus	CGHC/CGYC	197.21	1.60E-55	34.30
NP_593584.1	*Schizosaccharomyces pombe*	Fission yeast	CGHC/CGYC	190.66	2.61E-53	36.34
XP_013023086.1	*Schizosaccharomyces cryophilus*	Fission yeast	CGHC/CGYC	189.89	5.29E-53	35.71
WFD20523.1	*Malassezia caprae*	Opportunistic yeast	CGHC/CGFC	186.81	3.08E-51	32.41
XP_056064288.1	*Malassezia vespertilionis*	Cold-tolerant yeast	CGHC/CGYC	185.65	7.06E-51	30.73
XP_018741657.1	*Malassezia sympodialis*	Opportunistic yeast	CGHC/CGFC	185.27	8.71E-51	32.42
KAJ1929850.1	*Tieghemiomyces parasiticus*	Biotrophic mycoparasyte	CGHC/CGYC	183.34	2.82E-50	33.43
WFD23951.1	*Malassezia equina*	Opportunistic yeast	CGHC/CGFC	182.96	8.85E-50	31.49
XP_019025510.1	*Saitoella complicata*	Saprobic yeast	CGHC/CGYC	181.42	9.29E-50	33.92

WFD27832.1	*Malassezia nana*	Opportunistic yeast	CGHC/CGFC	181.03	4.90E-49	31.58
PWV20884.1	*Trypanosoma cruzi*	Parasitic protozoon	CGYC/CGHC	171.79	7.00E-46	31.78
XP_024664047.1	*Wickerhamiella sorbophila*	Budding yeast	CGHC/CGYC	171.01	1.15E-45	32.04
KAF3903448.1	*Dactylellina cionopaga*	Nematophagous fungus	CGHC/CGYC	168.70	1.14E-44	31.29
KFH67976.1	*Podila verticillata*	Soil fungus	CGHC/CGYC	164.08	4.95E-43	32.95
XP_012490347.1	*Gossypium raimondii*	New World cotton	CGHC/CGYC	161.00	5.05E-42	30.81
CAI0446640.1	*Linum tenue*	Flax	CGFC/CGHC	159.46	2.24E-41	31.03
KAF9304448.1	*Mortierella antarctica*	Endophytic fungus	CGHC/CGYC	159.84	2.35E-41	33.72
KAF8952714.1	*Haplosporangium bisporale*	Mucoromycotan fungus	CGHC/CGYC	163.70	3.01E-40	32.95
OLY81983.1	*Smittium mucronatum*	Endosymbiotic fungus	CPHC/CGFC	149.06	4.66E-37	30.41
PVV03876.1	*Smittium megazygosporum*	Endosymbiotic fungus	CGYC/CGFC	138.27	4.67E-32	30.43
KAG9061599.1	*Linnemannia hyalina*	Cold-adapted nitrogen reducing fungus	CGYC/CGYC	137.50	8.41E-32	32.50

Table S2. Electrospray ionization mass spectrometry analysis of full-length Erp41, *a*, *a'* wild type and *a'* W/F mutant. Both theoretical and experimental masses are reported as average masses. Theoretical average masses refer to the oxidized state of the proteins unless marked otherwise.

NEM	Protein	Theoretical Mass (Da)	Experimental Mass (Da)	#Disulfides (Da)	ΔMass (Da)	
-	Erp41	41188.89	41184.52	2	0.37	
	Erp41 wild type *a* domain	14010.97	14010.77	1	- 0.20	*
	Erp41 wild type *a´* domain	14343.20	14343.14	1	- 0.06	*
	Erp41 *a´* domain W/F	14304.16	14304.09	1	- 0.07	*
+	ERp41	41188.89	41184.02	4	0.87	
	ERp41 wild type *a* domain	14010.97	14261.01	1	-250.04	2 NEM
	ERp41 wild type *a´* domain	14343.20	14593.40	1	-250.198	2 NEM
	ERp41 *a´* domain W/F	14304.16	14554.35	1	-250.189	2 NEM

* Reduced Disulfide

Table S3. List of plasmids used in this study.

Plasmid	Background	Promoter	Expressed Gene
pMJS226	pLys	tac	Erv1p_hPDI (Classical CyDisCo) (53)
pAR_EC15	pLys	tac	Erv1p_KpPdi1 (Custom *Kp*PDI CyDisCo) (27)
pAR_EC35	pET23	tac	KpPdi1_KpEro1_6His
pAR_EC48	pET23	tac	KpErp41_6His
pAR_EC78	pET23	tac	KpErp41_KpEro1_6His
pAR_EC85	pET23	tac	KpErp41_*a*_6His
pAR_EC86	pET23	tac	KpErp41_*a'*_WT_6His
pAR_EC87	pET23	tac	KpErp41_*a'*_WF_6His
pAR143		-	Repair homology template
pAR145		pGAP/pLAT	sgRNA^{+}_Cas9
pAR149		pGAP/pLAT	sgRNA^{-}_Cas9

Table S4. List of oligonucleotides used in this study. Golden Gate fusion sites are underlined in the sequence.

Oligo	Sequence
sgRNA^{+}_Fw	atggtctcCCATGGAGCTGCTGATGAGTCCGTGAGGACGAAACGAGTAAGCTCGTCCAGC
sgRNA^{+}_Rv	AAACGAGTAAGCTCGTCCAGCTCAATGACCTCAGCAAgttttagagctagaaatagcaag
sgRNA^{+}_Fw	atggtctcCCATGGTAAAACTGATGAGTCCGTGAGGACGAAACGAGTAAGCTCGTCTTTT
sgRNA^{-}_Rv	AAACGAGTAAGCTCGTCTTTTACGTTGGCCTTTGCTGgttttagagctagaaatagcaag
Erp41_HR_dw_F	GATCTAGGTCTCAGGAGGTTGATGCCGTATTGGACG
Erp41_HR_dw_R	CAACCACTTGgagaccAGTAAATAAATAACGCTATAACTC
Erp41_HR_up_F	TTACTggtctcCAAGTGGTTGGACAGCAAAC
Erp41_HR_up_R	GTCATTGGTCTCTCATGGGTTTTTTTTTCCTGCCAGG
AR287_Erp41_KO_F	CTCTCATTGGGCTTCATCTAC
AR288_Erp41_KO_R	CCATAGTTTCAATCTGAAGTTGAC
qAR24_Erp41_up	GATTGCCGCCATTGATGCTA
qAR25_Erp41_mid	AGCCAGCTTGTTCATTGACA
qAR26_Erp41_mid	TCACAGCAGGCTCAGATGAA
qAR27_Erp41_dw	GCTCTTGGTGTAATAACTGGGG
